# The role of microRNAs in the pathogenesis of thyroid cancer

**DOI:** 10.1016/j.ncrna.2020.06.001

**Published:** 2020-06-20

**Authors:** Soudeh Ghafouri-Fard, Zeinab Shirvani-Farsani, Mohammad Taheri

**Affiliations:** a Department of Medical Genetics, Shahid Beheshti University of Medical Sciences, Tehran, Iran; b Department of Cellular and Molecular Biology, Faculty of Life Sciences and Technology, Shahid Beheshti University G.C., Tehran, Iran; c Urogenital Stem Cell Research Center, Shahid Beheshti University of Medical Sciences, Tehran, Iran

**Keywords:** Thyroid cancer, miRNA, Biomarker

## Abstract

Thyroid cancer is the most frequent type of cancers originating from the endocrine system. Early diagnosis leads to good clinical outcome in differentiated types of thyroid cancer. Yet, there are few treatment options for patients with medullary or anaplastic thyroid cancer. Thus, identification of molecular markers that explain the pathologic process during evolution of this cancer has practical significance. MicroRNAs (miRNAs) have been shown to influence the activity of thyroid cancer-related signaling pathways such as MAPK pathway and *RET* gene. These small transcripts not only can differentiate malignant tissues from non-malignant tissues, but also have differential expression in different stages of thyroid cancer. Assessment of serum levels of miRNAs is a practical noninvasive method for follow-up of patients after thyroidectomy. Moreover, the therapeutic effects of a number of miRNAs have been verified in xenograft models of thyroid cancer. In the current review, we summarize the data regarding the role of miRNAs in thyroid cancer.

## 1 Introduction

Thyroid cancer comprises the majority of tumors that originate from the endocrine system [1]. Based on the histological characteristics, thyroid cancers can be classified to differentiated thyroid cancer (DTC) originating from epithelial cells of the thyroid follicles, medullary thyroid cancer (MTC) and anaplastic thyroid cancer (ATC). Papillary thyroid cancers (PTCs) include most of DTCs. Other histological types of DTCs are follicular thyroid cancer (FTC) and Hürthle cells cancers [1]. Early detection of DTC and the appropriate surgical treatment and administration of radioiodine have improved prognosis of DTC. Yet, resistance to radioactive iodine is a major obstacle in the management of a proportion of patients with DTC. Besides, there are few treatment options for patients with MTC or ATC [1]. Thus, identification of molecular mechanisms for evolution of thyroid cancer is a necessity particularly for the management of histological subclasses that are less sensitive to the routine therapeutic options[2]. MicroRNAs (miRNAs) have recently attracted much attention for putative applications as tumor biomarkers and regulators of the carcinogenic process. Several studies have evaluated expression profiles of these ~20 nucleotide transcripts in thyroid cancer cell lines and clinical specimens. Based on their expression pattern in these tissues compared with non-malignant tissues and their effects on cell proliferation and apoptosis, miRNAs have been classified to oncogenic (oncomiRs) and tumor suppressor miRNAs. In the current review, we summarize the role of these transcripts in the pathogenesis of thyroid cancer and their possible application as biomarkers for thyroid malignancy.

## 2 OncomiRs in thyroid cancer

**Fig. 1:**
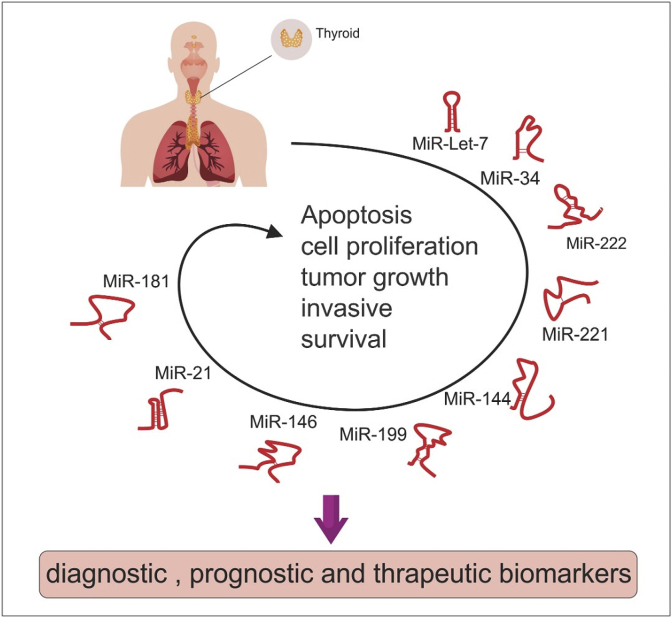
OncomiRs play important roles in the regulation of different processes in the thyroid cancer and can be used as diagnostic, prognostic, and therapeutic biomarkers in this cancer. These miRNAs induce cell proliferation and growth, invasion and metastasis, whereas, inhibit apoptosis. In addition, high expression of oncomiRs was related to a reduced survival rate.

*In vitro* and in vivo experiments have revealed the role of several miRNAs in the pathogenesis of thyroid cancer (Fig. 1). These oncomiRs have been shown to decrease expression of a number of tumor suppressors, thus enhancing cell proliferation and cell cycle progression. The role of these miRNA is exerted through modulation of cancer-related signaling pathways such as PI3K/Akt/mTOR, the adipocytokine signaling pathway, Hippo, Wnt and Jak-STAT signaling pathways.

Among the oncomiRs whose role in thyroid cancer have been assessed is miR-19a. This member of the miR-17-92 cluster is over-expressed in ATC tissues, promoting the de-differentiation and aggressiveness of the corresponding cells. Forced over-expression of this miRNA in the well-differentiated FTC cell line has enhanced cell proliferation and modified the signature of genes associated with thyroid cell differentiation and aggressiveness such as thyroid stimulating hormone receptor and thyroglobulin [3]. The oncogenic effects of the miR-223 in thyroid cancer cells are probably mediated through down-regulation of APQ-1 protein. Notably, siRNA-mediated silencing of this miRNA has inhibited cell proliferation and induced apoptosis in these cells [4]. Besides, miR-221 has been shown to directly bind with the 3′ untranslated region (3′UTR) of TIMP3, thus inhibiting its expression and promoting proliferation and invasion of PTC cells. The oncogenic effects of this miRNA has been also verified in xenograft model of PTC [5]. miR-222 has been identified as another oncomiR in PTC based on its over-expression on PTC patients compared with goiter group. Besides, its expression levels were higher in patients with larger tumor sizes and invasive properties. Expression of miR-222 was also correlated with the risk levels provided by the American Thyroid Association, but not with the TNM staging [6]. Expression of miR-181a has also been increased in thyroid cancer tissues compared with the paired non-cancerous tissues. Functional studies showed that miR-181a silencing decreases cell growth, while its up-regulation inhibits apoptosis and enhances cell cycle progression. This miRNA inhibits expression of RB1 [7]. Another study has demonstrated up-regulation of miR-146b, miR-222, miR-21, miR-221 and miR-181b in PTC tissue samples compared with normal thyroid tissues. Over-expression of these miRNAs were also detected in recurrent PTC tumors compared with non-recurrent samples and in lymph node metastases (LNM)-positive samples compared LNM-negative ones. Yet, distribution expression levels of these miRNAs were not different between PTC patients that have high and low risk of recurrence [8]. Expression of miR-146b-5p, miR-146b-3p, miR-221-3p, miR-222-5p, miR-222-3p has been increased in PTC tissues compared with normal thyroid samples. These were significant associations between up-regulation of miR-146b-5p and miR-222-3p and higher risk of recurrence. Over-expression of miR-146b-5p and miR-146b-3p distinguishes classical type and tall-cell variant but not follicular variant of PTC. Besides, miR-21-5p was remarkably increased only in tall-cell variant. Therefore, expression profile of miRNAs might be used in the molecular classification of PTC [9]. Table 1 summarizes the function and molecular interactions of oncomiRs in thyroid cancer.

**Table 1:** OncomiRs which are up-regulated in thyroid cancer.

microRNA	Numbers of clinical samples	Assessed cell line	Targets/Regulators	Signaling Pathways	Function	Ref
miR-19a	–	FTC-133, 8505c	PTEN, TSHr, Tg, TTF1 and Pax8, CDH1, an E-cadherin	–	miR-19a overexpression stimulates cell proliferation and alters the expression signature of genes associated with thyroid cell differentiation and aggressiveness.	[3]
miR-222	10 patients with multinodular goiter and 90 with PTC	–	–	–	miR-222 expression was correlated with ATA risk levels.	[6]
miR-223	–	SW579, Nthy-ori3-l	APQ-1	–	miR-223 inhibitor suppresses proliferation and activates apoptosis of thyroid cancer cells by down-regulating AQP-1.	[4]
miR-34a	28FFPE MTC samples along with ANTs	–	AXL	PI3K/Akt/mTOR	miR-34a suppresses the expression and functions of AXL and impair migration, invasion, and formation of distant metastasis.	[10]
miR-144	28FFPE MTC samples along with ANTs	–	mTOR	PI3K/Akt/mTOR	Its repression decreases cell proliferation, clonogenicity, migration, invasion, and tumor formation in animal model.	[10]
miR-181a	15 paired thyroid cancer tissues and ANTs	8505C, SW1736, TPC-1, Nthy-ori3-1	RB1	–	miR-181a overexpression decreased apoptosis and promoted cell cycle progression	[7]
miR-221	The PTC biopsy specimens (n = 65)	TPC-1, BCPAP, HEK293T	TIMP3	–	miR-221 could aggravate cell proliferation and invasion by targeting TIMP3.	[5]
miR-375	thyroid tissue s from130 patients affected by MTC (104 sporadic and 26 familial)	–	YAP1	AKT	miR-375 plays an essential role in MTC progression.	[11]
miR-146a and miR-146b	73 PTC tissues and ANTs	–	IRAK1	TLRs/IL-1	Expression levels of miR-146a and miR-146b influence the cell proliferation and migration.	[12]
miR-375	plasma from 37 MTC patients with persistent or recurrent metastatic disease, 9 non-metastatic MTC patients in remission and 36 HCs	–	YAP1, SEC23A	PI3K/Akt	Deregulation of miR-375 participates in MTC tumorigenesis. Circulating miR-375 is as an independent prognostic marker for metastatic MTC.	[13]
miR-9-3p	Frozen biopsy specimens from 12 patients with MTC and eight non-tumor donors	TT cells	BLCAP	Bcl-XL/Bcl-2	Upregulated miR-9-3p has a positive role in human MTC progression by modulating the growth and apoptosis of cancer cells.	[14]
miR-205	–	MB-1 and BHT-101	VEGF-A, ZEB1	–	Up-regulation of miR-205 significantly suppressed angiogenesis.	[15]
miR-340-5p	49 cancer samples and 20 relatively normal samples	HT-ori3, SW579 and NPA	BMP4	–	miR-340-5p promotes thyroid cancer proliferation.	[16]
let-7	Plasma from 49 PTC, 21HC	–	–	–	Abnormal expression of let-7 has been associated with cancer initiation and progression.	[17]
miR-222	Five PTC tumor samples and ANTs	–	–	the adipocytokine signaling pathway and Jak-STAT signaling pathway	miR-222 may play critical roles in tumorigenesis of PTC.	[18]
hsa‐miR‐181a‐2‐3p	32 pairs of PTC and ANTs	–	–	–	This miRNA signature could predict survival of patients with PTC.	[19]
miR-221	30 PTC cancer samples and ANTs	TPC-1, K1 and BCPAP, Nthy-ori 3-1	RECK	–	miR-221 promoted the proliferation, migration and invasion activities of PTC K1 cells.	[20]
miR-222	Blood from 38 PTC patients and 30 HCs	–	PPP2R2A	AKT	Causing more aggressive behavior of the tumor	[21]
miR-155	Blood from 38 PTC patients and 30 HCs	–	–	–	Causing more aggressive behavior of the tumor	[21]
miR-146b-5p	7 PTC tumors and contralateral normal thyroid tissue	BCPAP and TPC1, Ocut2, Ktc2, Cal62, T235, Hth83, Hth74, and SW1736	DICER1	DICER1 pathway	miR-146b increases proliferation, migration, and invasion.	[22]
miR-222-3p, miR-17-5p, and miR-451a	Serum from 295 participants including 100 patients with PTC, 91 patients with benign nodules, 15 patients with MTC, and 89 HCs	–	–	–	miR-222-3p, miR-17-5p and miR-451a might discriminate PTC and benign thyroid nodules from controls. miR-222-3p and miR-17-5p serum levels may be biomarkers for differential diagnosis of MTC from benign thyroid nodules.	[22]
hsa‐mir‐6843, hsa‐mir‐6730	491 PTC tissues and 59 ANTs			Hippo signaling pathway, proteoglycans in cancer, axon guidance, Wnt signaling	These miRNAs were identified as potential prognostic predictors of the 5‐year survival and OS in patients with PTC.	[23]
miR-146b, miR-222, miR-21, miR-221 and miR-181b	400 FFPE PTC tissue specimens and ANTs	–	–	–	The levels of miRNA-146b, - 222, −21, −221 and −181b expression in PTC were strongly associated with PTC recurrence and lymph node metastases.	[8]
miR-146b-5p, miR-146b-3p, miR-221-3p, miR-222-5p, miR-222-3p	76 normal and neoplastic thyroid tissues from 29 PTC patients	–	–	–	Dysregulated expression of several miRNAs that distinguish these cancers from normal thyroid tissue.	[9]
miR-146-5p	56 normal and neoplastic thyroid tissues from 507 PTC patients	–	TRAF1 and PML	cancer, apoptosis, and calcium signaling pathways	miR-146b-5p may play an essential role in the progression of PTC and influence the biological processes of cancer cells.	[24]
miR-146a-5p and miR-221-3p	Serum from 44 patients with sporadic PTCs and 39 controls				Serum levels of miR-146a-5p and miR-221-3p are biomarkers for the early noninvasive detection of persistent/recurrent PTC.	[25]
miR-221, miR-222, miR-146b, miR-34a, miR-144	499 PTC samples and 58 normal thyroid tissues	–	AXIN2, BCL2, RUNX1, CCNE2,	–	These miRNAs have potential clinical applications for diagnosis, prognosis, and targeted treatment in thyroid malignant disease.	[26]
miR‐221‐5p, miR‐222‐5p, miR‐34a‐5p, miR‐146b‐5p, miR‐21‐5p, miR‐31‐5p	25 PTC samples and ANTs	–	–	–	The identified miRNAs may be potential diagnostic/prognostic biomarkers and therapeutic targets.	[27]
miR-146b	48 samples from paired PTC tumors and ANTs	A549, HeLa, K1 cell line	RARB	–	miR-146 Family increases Proliferation of the PTC-Derived Cell Line	[28]
miR-515, miR-192	127 thyroid tumors (26 were follicular adenomas, 23 follicular carcinomas, and 78 PTC) and 17 normal thyroid tissues	–	–	–	Deregulated miRNAs play roles in the development of well-differentiated thyroid cancer and are novel markers associated with recurrence-free survival.	[29]
miR-375	Tissues from 62 MTC patients	Nthy-ori 3-1, TT cells, 8505C, B-CPAP	SEC23A	ERK, AKT pathways	Expression of miR-375 in Nthy-ori 3-1 cells decreased cell proliferation after with an increase in the percentage of cells in G1 miR-375 increased mortality.	[30]
miR-182	30 pairs of ATC and ANTs	SW1736, 8305C, and Nthy-ori 3-1	TRIM8	–	miR-182 enhances cellular growth by repressing TRIM8 expression.	[31]
miR-23a	Twenty paired tissue specimens of human PTC and ANTs	K1 cells	PTEN		miR-23a enhances cell proliferation and invasion and suppresses apoptosis of PTC cells by targeting PTEN.	[32]
miR-146b	71 paired tissue specimens of human PTC and ANTs	BCPAP	–	–	miR-146b is a novel prognostic biomarker of PTC.	[33]

(HC: healthy control, PTC: papillary thyroid carcinoma, ANT: adjacent normal tissue, MTC: medullary thyroid cancer, ATC: anaplastic thyroid cancer, FTC: follicular thyroid cancer HC: healthy control).

## 3 Tumor suppressor miRNAs in thyroid cancer

Several miRNAs have been shown to negatively regulate expression of oncogenes, thus inhibiting cell proliferation and migration. MAPK, PI3K, NF-κB, GSK-3β/β-catenin, AKT and PI3K pathways are among cancer-related pathways which are modulated by these miRNAs. An extensive number of these miRNAs have been shown to be down-regulated in thyroid cancer cell lines or clinical samples, thus facilitating malignnat behavior of these cells. For instance, miRNome sequencing has shown constant down-regulation of hsa‐miR‐139‐5p in patients with recurrent or metastatic thyroid cancer compared to disease‐free patients. Functional studies have shown the roel of this miRNA in attenuation of cell migration and proliferation in ATC cells. RICTOR, SMAD2/3 and HNRNPF have been identified as pssible targets for this miRNA. Moreover, expression of hsa‐miR‐139‐5p has been inversely correlated with the expression of *HNRNPF* transcript, which codes for an alternative splicing factor participating in cryptic exon inclusion/skipping [34]. Besides, miR-128 has been shown to target sphingosine kinase-1 (SPHK1) thrugh direct interaction with its 3′UTR. Over-expressiion of this miRNA has led to attenuation of tumor growth rate and tumor weight in tumor-bering animals [35]. Up-regulation of miR-let-7e has been shown to suppress cell migration and invasion of thyroid cancer cells. This miRNA inhibits HMGB1 expression through binding with its 3′ UTR. miR-let-7e has been regarded as a tumor suppressor miRNA in PTC and a putative therapeutic candidate for this kind of cancer [36]. miR-129 is another tumor suppressor miRNA in PTC which exerts its function through inhibition of expression. Over-expression of miR-129 inhibits growth and invasion of PTC cells. Thus, miR-129-MAL2 axis is regarded as a therapeutic target in PTC [37]. Expression of miR-26b-5p has been decreased in thyroid cancer tissues compared with adjacent normal tissues in association with lymph node metastasis. In vitro studies showed the role of this miRNA in suppression of cell proliferation, invasion and migration of thyroid cancer cells. The tumor suppressor role of this miRNA might be exertedv through the Gsk-3β/β-catenin pathway [38]. miR-203 has been down-regulated in PTC tissues and cell lines compared with control tissues and cells. Down-regulation of this miRNA was associated with up-regulation of survivin, through which miR-203 modulates Bcl-2 expression [39]. Table 2 summarizes the functions and molecular interaction of the tumor suppressor miRNAs in thyroid cancer.

**Table 2:** Tumor suppressor miRNAs in thyroid cancer.

microRNA	Numbers of clinical samples	Assessed cell line	Targets/Regulators	Signaling Pathways	Function	Ref
hsa-miR-139-5p	a fresh frozen thyroid tissue series including 3 normal tissues, 4 adenomas and 42 carcinomas	CAL-62 and 8505C	RICTOR, SMAD2/3 and HNRNPF	MAPK and PI3K	hsa-miR-139-5p/HNRNPF expression modulates the transcript balance of genes participating in important cancer-related signaling pathways.	[34]
miR-128	30 pairs of primary PTC (24 cases) and FTC (6 cases) tissue specimens and ANTs	FTC-133, FTC-236, TPC-1, CAL-62, FRO, ARO and K1, Nthyori3-1	SPHK1, Bmi-1, EGFR and E2 F3	–	Over-expression of miR-128 decreased cancer cell viability, activated apoptosis and cell arrest in G0/G1 phase.	[35]
miR-let-7e	–	Male athymic BALB/c nu/nu mice, BCPAP and TPC-1 cell lines	HMGB1	NF-κB	Overexpression of miR-let-7e suppresses PTC cell migration and invasion.	[36]
miR-129	48 pairs of PTC tissues and ANTs	BCPAP, KTC-1, TPC-1 and K1, Nthy-ori3-1	MAL2	–	miR-129 inhibits growth and invasion of PTC cells by targeting MAL2.	[37]
miR-214	Human clinical PTC tissues from 30 patients and ANTs	CGTH W-3 and PTC- uc3, Nthy-ori 3-1	PSMD10	GSK-3β/β-catenin and AKT signaling	Upregulation of miR-214 reduced cell proliferation, and enhanced cell apoptosis and cell cycle arrest in PTC cell lines.	[40]
miR-34a	A total of 77 paired thyroid cancer and non-tumor tissue samples	FTC133, BCPAP, TEC, TPC-1, SW1736, KAT18	MET, XIST	PI3K and AKT	XIST negatively interacts with miR-34a to regulate cell proliferation and tumor growth.	[41]
miR-206	Tissue samples of 23 patients and ANTs	Nthy-ori3-1, PTC cell line TPC-1	MAP4K3	p38 and JNK	miR-206 suppressed cell proliferation, enhanced apoptosis, reduced the expressions of multidrug resistance-related proteins	[42]
miR-335-5p	Surgical resection of thyroid cancer and ANTs	TPC-1, FTC-133, TT, Nthyori 3-1	ICAM-1	–	inhibit the proliferation of thyroid cancer cells	[24]
miR-199b-5p	40cases of PTC tissues and eight cases of ANTs	SW579 and B-CPAP	STON2	–	Overexpression of miR-199b-5p inhibited cell proliferation, promoted apoptosis.	[43]
miR-718	15 pairs of PTC and ANTs	TPC-1,K1and 293T	PDPK1	Akt-mTOR	miR-718 negatively controls PTC cell proliferation, migration, and invasion.	[44]
miR-429	59 thyroid cancer and ANTs	Nthy-ori 3–1, TCP-1 and NPA	ZEB1	–	miR-429 suppressed cell proliferation, migration and invasion	[45]
miR-26b-5p	67 TC tissues and 67 ANTs	BC-PAP	Gsk-3β and β-catenin	Gsk-3β/β-catenin	miR- 26b-5p overexpression suppresses cell proliferation, migration and invasion.	[38]
miR-381-3p	53 Fresh frozen tissues from PTC patients, 24 normal thyroid tissues	TPC-1, BCPAP, K1, Nthyori3-1	LRP6	–	Up-regulation of miR-381-3p inhibits PTC cell proliferation, migration and invasion.	[46]
miR-524	fresh cancer tissues ANTs	WRO, TPC1	SPAG9	–	Up-regulated miR-524 expression suppressed the proliferative ability and promoted cell apoptosis.	[47]
miR-9	60 pairs of fresh frozen PTC tissue samples and ANTs	TPC-1	BRAF	MAPK	miR-9 may suppress the viability of PTC cells and inhibit tumor growth.	[48]
miR-205	132 paired thyroid carcinoma and ANTs	8505-C, BCPAP, BHT-101	YAP1	hippo	miR-205 inhibited certain aspects of thyroid cancer, including cell proliferation, migration and invasion.	[49]
MiR-431	Sixty-six PTC patient tissue samples and 38 ANTs	PTC-1 and BCPAP	E-cadherin, Vimentin	Hedgehog	miR-431 inhibited cell migration and invasion.	[50]
miR-486-5p	507 PTC and 59 ANTs	–	FBN1, CRKL, PTEN and TPM3	hsa05200	a clinical biomarker for PTC	[51]
miR-577	35 PTC tissues and matched ANTs	TPC-1, BCPAP, K1, Nthy-ori3-1	SphK2	–	Up-regulation of miR-577 inhibited the proliferation, migration and invasion of PTC cells.	[52]
miR-125b	30 paired Tumor specimens and ANTs	SW1736, 8305C, Nthy-ori3-1	PIK3CD	PI3K/Akt/mTOR	miR-125b represses migration and invasion.	[53]
miR-132	30 paired human thyroid cancer specimens and ANTs	TPC1, GLAG-66, Nthy-ori 3-1	FOXA1	–	Overexpression of miR-132 in TPC1 cells inhibited cell proliferation, migration, and invasion.	[54]
miR-212	42 primary thyroid cancer samples and ANTs	TPC-1, BCPAP and SW1736, Nthy-ori3-1	SIRT1	–	miR-212 overexpression significantly inhibited tumor growth	[55]
miR-217	58 paired thyroid cancer tissues and ANTs	8505C, TPC-1, and SW1736, Nthy-ori3-1	AKT3	–	miR-217 overexpression inhibited proliferation, migration, and invasion.	[56]
miR-199a-3p	188 tissue samples (136 PTCs, 52 normal thyroid tissue)	–	–	–	miR-199a-3p activation in PTC cells suppresses migration and proliferation.	[57]
miR-199a-5p	24 pairs of primary PTC tissue specimens and ANTs	TPC-1and K1 and HEK 293T, Nthy-ori3-1	SNAI1	–	miR-199a-5p overexpression suppressed tumor growth.	[58]
miR-150	Ten pairs of thyroid tissues, consisting of human thyroid cancer tissue and ANTs	K1 and TPC-1	RAB11A	WNT/b-catenin	Overexpression of miR-150 suppressed cell proliferation via inducing the cell cycle arrest and promoting cell apoptosis.	[59]
miR-144	59 paired PTC tissues and ANTs	BCPAP and TPC-1	E2F8	–	miR-144/E2F8/CCND1 regulatory axis controls PTC development.	[60]
miR-211-5p	Forty pairs of the thyroid cancer and ANTs	K1/BCPAP/TPC-1, Nthy-ori3-1	SOX11	–	MiR-211-5p affected the viability, proliferation and invasion of TC.	[61]
miR-135a-5p	Fifty-three pairs of human thyroid carcinoma and ANTs	FTC-133, TPC1 and K1, STC, SW579, HT-ori3	VCAN	–	miR-135a-5p could affect the proliferation, invasion and migration of thyroid carcinoma cells.	[52]
miR-7-2	Five PTC tumor samples and ANTs	–	CLDN1	tight junction pathway	miR-7-2 and CLDN1 may be used as biomarkers of stage and prognosis in PTC.	[18]
miR‐153‐3p	–	The human MTC TT cell line	RPS6KB1	mTOR	miR‐153‐3p acts as a tumor suppressor in MTC tumorigenesis.	[62]
hsa‐miR‐138‐1‐3p	32 pairs of PTC and ANTs	–	–	–	this miRNA signature could independently predict the survival of patients with PTC.	[18]
miRNA-564	Paired PTC and ANTs obtained from 47 patients	TPC-1, BCPAP, and HTH83, HT-ori3	AEG-1	PTEN/Akt	miR-564 upregulation suppressed cell proliferation, migration, and invasion and induced cell apoptosis.	[63]
miRNA-384	58 cases of PTC and their ANTs	BCPAP, K1	PRKACB	PKA signal transduction pathway	miR-384 is a tumor suppressor that targets the 3′-UTR of PRKACB gene.	[64]
miR-203	30 cases of PTC and ANTs	Nthy-ori3-1, HTH83, NIM-1 and TPC-1	Survivin	–	miR-203 inhibits cell proliferation and migration, and enhances apoptosis.	[39]
miR-146a-5p, miR-132-3p, and miR-183-3p	Serum from 295 participants including 100 patients with PTC, 91 patients with benign nodules, 15 patients with MTC, and 89 HCs	–	–	–	miR-146a-5p, miR-132-3p, and miR-183-3p might be biomarkers for discrimination of PTC and benign thyroid nodules from controls.	[22]
hsa‐mir‐196a‐2, and hsa‐mir‐206	491 PTC tissues and 59 ANTs	–	–	Hippo signaling pathway, proteoglycans in cancer, axon guidance, Wnt signaling	These miRNAs are potential prognostic predictor of the 5‐year survival and OS in patients with PTC.	[23]
hsa-miR-146b, hsa-miR-146b, hsa-miR-222, hsa-miR-221, hsa-miR-134, hsa-miR-34a, hsa-miR-101, hsa-miR-143, hsa-miR-144, hsa-miR-615, hsa-miR-375, hsa-miR-181b, hsa-miR-194, hsa-miR-130a, hsa-miR-199a-3p, hsa-miR-30a, hsa-miR-424, hsa-miR-148a, hsa-miR-24	102 TC tumors and contralateral normal thyroid tissue patients				These 19 miRNAs may be used to discriminate benign from malignant thyroid nodules.	[65]
miR-1179, miR-486-5, miR-204-5p, miR-7-2-3p, miR-144-5p, miR-140-3p	76 normal and neoplastic thyroid tissues from 29 PTC patients	–	–	–	Dysregulated expressions of these miRNAs distinguish these cancers from normal thyroid tissue.	[9]
miR-138/miR-21	101 PTC and 51 benign thyroid nodule (control) patients	–	–	–	miR-138 expression was not only associated with onset of PTC, but also the aggressiveness of PTC. Combination of miR-138 and miR-21 could increase the diagnostic accuracy for PTC.	[66]
let-7b	20 pairs of PTC tissues, and ANTs, and 10 cases of adjacent thyroid benign lesions	BCPAP, IHH4, TPC-1, CGTHW-3	HMGA2	–	Let-7b overexpression inhibited cell proliferation, migration, and invasion. Let-7b suppressed in vivo tumor growth.	[67]
miR‐181‐5p, miR‐138‐5p	Twenty‐five PTC samples and ANTs	–	–	–	The identified microRNAs may be potential diagnostic/prognostic biomarkers and therapeutic targets.	[27]
miR-4728	18 pairs of PTC and ANTs	TPC-1, K1 and Nthy-ori 3-1	SOS1	MAPK signaling pathway	miR-4728 suppresses human PTC cell proliferation, miR-4728 suppresses MAPK signaling pathway.	[68]
miR-1247, let-7a	127 thyroid tumors (26 were follicular adenomas, 23 follicular carcinomas, and 78 PTC) and 17 normal thyroid tissues	–	–	–	Deregulated microRNAs play roles in the development of well-differentiated thyroid cancer and are novel markers associated with recurrence-free survival.	[29]
miR-451	Tissues from 62 MTC patients	–	–	–	miR-451 decreases cell proliferation.	[30]
miR-215	Forty-eight pairs of human PTC and ANTs	Nthy-ori 3–1, TPC-1, K1, BCPAP, IHH4	ARFGEF1	AKT/GSK-3β/Snail signaling	miR-215 suppresses PTC proliferation, migration, and invasion through the AKT/GSK-3β/Snail axis by targeting ARFGEF1. It was negatively associated with prognosis in patients with PTC.	[69]
miR-125b	Thirty pairs of thyroid samples, consisting of tumor and non-tumor tissues	Human FTC, ATC, and Nthy-ori 3-1, Nthy1	Foxp3	Atg7 pathway	miR-125b promotes autophagy in thyroid cancer cells through Atg7.	[70]
miR-23a	28 paired of PTC tissue samples and ANTs	PTC cell lines	CCNG1	–	Upregulation of miR-23a reduces cell proliferation, induced cell cycle arrest at G0/G1 phase and stimulated cell apoptosis.	[71]

(PTC: papillary thyroid carcinoma, ANT: adjacent normal tissue, MTC: medullary thyroid cancer, ATC: anaplastic thyroid cancer, FTC: follicular thyroid cancer).

## 4 Diagnostic/prognostic role of miRNAs in thyroid cancer

Several studies have assessed diagnostic accuracy of miRNAs in thyroid cancer. Among them is the study conducted by Rosignolo et al. which identified serum profile of 754 miRNAs in PTC patients prior to and after thyroidectomy [25]. Notably, expression of eight miRNAs was significantly higher in patients before treatment compared with their levels both in healthy subjects and afer-treatmnet samples. The most promising results were reported for miR-146a-5p and miR-221-3p. Thus, expression of these miRNAs can be used as biomarkers for follow-up of patients. Prognostic significance of miRNAs in thyroid cancer has been verified through application of Kaplan-Meier analysis and cox regression methods. For instance, Wen et al. have reported consistent down-regulation of miR-486-5p in a number of PTC samples from TCGA, GEO and ArrayExpress datasets. They also reported associations between expression levels of this miRNA and clincal parameter such as cancer stage, lymph node involvement, distant metastsis and most notably overall survival [51]. Mazel et al. have assessed miRNA profiles in thyroid samples using next generation sequencing and multiplexing technologies. They recognized significant differences in miRNA signature between normal and malignant tissues. Notably, expression of 19 miRNAs were significantly different between benign and malignant tissues. In the bvalidation step, these miRNAs could classify 35 other nodules with indeterminate cytology. This panel has sensitivity, specificity and diagnostic power of 91%, 100% and 94%, respectively, which are superior to the existing molecular assays [65]. Table 3 summarizes the results of studies which appraised diagnostic/prognostic significance of miRNAs in thyroid cancer.

**Table 3:** Diagnostic/prognostic role of miRNAs in Thyroid cancer.

Sample number	Area under curve	Sensitivity	Specificity	Kaplan-Meier analysis	Univariate cox regression	Multivariate cox regression	Ref
a fresh frozen thyroid tissue series including 3 normal tissues, 4 adenomas and 42 carcinomas	–	–	–	DFS analysis showed significant differences in the time to relapse or death based on expression levels of hsa-miR-139-5p in DTC patients.	–	–	[72]
102 TC tumors and contralateral normal thyroid tissue patients	0.95	91%	100%	–	–	–	[65]
76 normal and neoplastic thyroid tissues from 29 PTC patients	–	–	–	–	–	Nine miRNAs were associated with a higher risk of tumor recurrence. The expression of miR-146b-5p and miR-222-3p was upregulated in intermediate-risk PTCs as compared to low-risk tumors.	[9]
56 normal and neoplastic thyroid tissues from 507 PTC patients	0.91	–	–	Patients in advanced stages showed higher levels of miR-146b than those in early stages. Moreover, cases with extrathyroid extension also had markedly higher levels of miR-146b compared to those without.	–	–	[24]
101 PTC and 51 benign thyroid nodule (control) patients	0.71 for miR-138, 0.61 for miR-21	84% for miR-138, 76% for miR-21	49.5% for miR-138, 51% for miR-21	–	miR-138 expression was down-regulated in PTC with aggressive features, including advanced tumor stage (stage III, IV), capsule invasion, lymph node metastasis and extrathyroidal extension, only with statistical significance in PTC with capsule invasion.	miR-138 had a protective role and miR-21 had a predictive value for PTC	[66]
Serum from 44 patients with sporadic PTCs and 39 controls	0.9 for miR-146-5p, 0.93 for miR-221-3p, 0.85 for miR-222-3p	79.5% for miR-146-5p, 88.6% for miR-221-3p, 90% miR-222-3p	52% for miR-146-5p, 100% for miR-221-3p, 84.2% miR-222-3p			miR-146a-5 and miR-221-3p had good accuracy in discriminating between PTC patients and controls.	[25]
499 PTC samples and 58 normal thyroid tissues	0.961 for miR-221, 0.958 for miR-222, 0.95 for miR-146b, 0.944 for miR-34a, 0.924 for miR-144	91.4% for miR-221, 91.4% for miR-222, 84.5% for miR-46b, 91.4% for miR-34a, 94.8% for miR-144	96.6% for miR-221, 94.8% for miR-222, 96.6% for miR-46b, 94.8% for miR-34a, 81% for miR-144	–	The expression levels of miR-146b, miR-222, miR-221, miR-34a were associated with PTC invasion or progression. miR-146b and miR-222 were associated with all high-risk clinical features.	Several miRNA and target combinations improved PTC diagnosis accuracy.	[26]
127 thyroid tumors (26 follicular adenomas, 23 follicular carcinomas, and 78 PTCs) and 17 normal thyroid tissues	–	–	–	Increased expression of let-7a, together with decreased miR-192 expression, was associated with an increased risk of recurrence.	Relapse prediction model was based on expression of let-7a, and miR-192 and several other clinicopathological features.	–	[29]
491 PTC tissues and 59 corresponding normal tissues	0.886	–	–	Poor OS was found in the patients in the high‐risk group than in those in the low‐risk group for all the patients and subclasses.	–	The miRNA signature was an independent prognostic factor associated with OS.	[73]
40 PTC tissues and eight ANTs	–	–	–	Patients with a lower miR‐199b‐5p level exhibited a shorter survival, and patients with higher miR‐199b‐5p expression had a longer survival time.	–	–	[43]
28FFPE MTC samples along with ANTs	0.95 for miR-34, 0.95 for miR-144	89% for miR-34, 93% for miR-144	80% for miR-34, 80% for miR-144	–	–	–	[74]
507 PTC and 59 normal thyroid samples	0.91	–	–	The median OS for the high expression group was 1,443 days, whereas the median OS for the low expression group was 1,015 days. The curves suggested that PTC cases with higher miR-486-5p expression levels were likely to have an improved clinical outcome.	–	–	[51]
188 tissue samples (136 PTCs, 52 normal thyroid tissue)	0.87	–	–	–	Low miR-199a-3p expression levels were linked to TNM stage (p = 0.026), extra-thyroidal extension (p = 0.02), lymph node (LN) metastasis (p = 0.036), distant metastasis (p = 0.002) and recurrence of LN metastasis	–	[57]
73 PTC tissues and ANTs	–	–	–	It was observed that the survival time of the patients with high expression of miR-146a and miR-146b was significantly shorter than that of the patients in the normal or low expression groups	–	–	[75]
plasma from37 MTC patients with persistent or recurrent metastatic disease, 9 non-metastatic MTC patients in remission and 36 HCs	0.88	86.1%	88.9%	Patients with higher levels of miR-375 had a striking and significantly worse OS.	Poor prognosis was associated only with male sex, tumor burden and high plasmatic levels of miR-375.	Only high levels of miR-375, but not male sex nor tumor burden, maintained the prognostic significance of worse outcome.	[13]
Plasma from 49 PTC, 21HC	0.66	74%	38%	–	–	–	[17]
Forty-eight pairs of human PTC and ANTs	–	–	–	Patients with lower miR-215 expression exhibited significantly DFS than patients with higher miR-215 expression.	Downregulation of miR-215 expression was negatively associated with tumor size, differentiation, and lymph node metastasis status.	–	[69]
71 paired tissue specimens of human PTC and ANTs	–	–	–	Patients with primary tumors expressing higher miR-146b levels had a lower DFS rate than those with lower miR-146b expressions.	–	miR-146b expression was a prognostic factor for DFS rate in patients with PTC. Advanced tumor stages and cervical LN metastasis were poor prognostic factors of DFS in patients with PTC at follow-up.	[33]

(ANT: adjacent normal tissue, OS: overall survival, RFS: relapse-free survival, DFS: disease-free survival, PTC: papillary thyroid carcinoma, HC: healthy control, DTC: differentiated thyroid cancer, MTC: medullary thyroid cancer, LN: lymph node).

## 5 Role of miRNAs in chemoresistnce in thyroid cancer

The significance of miRNAs in determination of response to anti-cancer agents has been addressed in thyroid cancer patients. For instance, the tumor suppressor miRNA, miR‐199b‐5p has been shown to enhance sensitivity of thyroid cancer cells to the chemotherapeutic agent paclitaxel [43]. Moreover, miR-125b has significantly sensitized thyroid cancer cells to the effcts of cisplatin by activating autophagy through an Atg7 dependent route [70]. Most notably, miR-375 expression levels has been associated with reduced cell proliferation and improved sensitivity to vandetanib, a multi-kinase inhibitor which is used as a therapeutic option for metastatic MTC [30]. Table 4 summarizes the results of studies which reported association between expression levels of miRNAs and response to anti-cancer drugs.

**Table 4:** Role of miRNAs in response to anti-cancer drugs in thyroid cancer.

Response to anti-cancer drug	miRNA	Function	Reference
Paclitaxel sensitivity	miR‐199b‐5p	Up-regulation of miR-199b-5p suppresses cell proliferation, enhances apoptosis, and improves the sensitivity of thyroid carcinoma cells to paclitaxel. This miRNA inhibits tumor growth in nude mice.	[43]
Cisplatin resistance	miR-182	miR-182 enhances cell growth through suppressing TRIM8 expression. Up-regulation of miR-182 enhances resistance of ATC cells to cisplatin by the suppression of TRIM8.	[31]
Cisplatin sensitivity	miR-125b	Up-regulation of miR-125b enhances sensitivity of thyroid cancer cells to cisplatin through regulation of autophagy.	[70]
Vandetanib sensitivity	miR-375	Up-regulation of miR-375 has reduced cell proliferation and synergistically enhanced sensitivity to vandetanib.	[30]
Resistance to chemotherapy	miR-146b	Up-regulatiion of miR-146b promotes cell migration and invasive features. This miRNA confers resistance to chemotherapy-induced apoptosis.	[33]

## 6 Discussion

Recent studies have revealed aberrant expression of miRNAs in tissues or peripheral blood of patients with thyroid cancer. These miRNAs have been involved in the regulation of signaling pathways such as MAPK, PI3K, AKT, GSK-3β/β-catenin, Wnt, mTOR and NF-κB. Recent studies have revealed association between DTC and mutations in the RAS/RAF/MAPK pathway or *RET/PTC* rearrangements [1]. Moreover, MTC tumors have been linked with activating mutations in the *RET* gene [1]. The observed dysregulation of MAPK-associated miRNAs in thyroid cancer further shows the complex interactive network between miRNAs and signaling pathways in the context of thyroid cancer. Few studies have shown association between RET and miRNAs in this kind of cancer. For instance, miR-153-3p has been shown to be a RET-regulated tumor suppressor miRNA in MTC [62]. Besides, expression of the oncogenic miR-182 has been increased in RET mutated cells. Notably, suppression of RET oncogenic signaling has decreased expression of miR-182. RET induced NF-κB translocation also affects expression of this miRNA. Notably, a known suppressor of the Notch pathway is targeted by miR-182 in mutant RET cell lines [76]. Therefore, miRNAs may serve as functional links between several cancer-related pathways in thyroid cancer.

The possibility of application of miRNA-targeted therapies in thyroid cancer has been assessed in some animal studies. For example, targeted intravenous transport of miR-153-3p has suppressed tumor growth in a xenograft model of MTC. This therapeutic option has been shown to have synergic effects with the tyrosine kinase inhibitor cabozantinib as well [62]. Thus, miRNA-targeted therapies might also reverse resistance to other anti-cancer therapies.

Diagnostic power of miRNAs in thyroid cancer has been evaluated by several groups. miRNAs not only can differentiate malignant tissues from non-malignant tissues, but also have differential expression in different stages of thyroid cancer. Assessment of serum levels of miRNAs is a practical noninvasive method for follow-up of patients after thyroidectomy. Notably, a transcript signature consisting of 19 miRNAs could discriminate benign lesions from malignant thyroid nodules with unknown cytology at better accuracy and lower expense compared with existing molecular assays [65]. However, diagnostic power of these panels of miRNAs should be appraised in different populations to obtain the best panel for each ethnic group. It is worth mentioning that the presence of single nucleotide polymorphisms in both miRNAs and the mRNA targets might alter their bindings. Thus, the significance of each oncomiR or tumor suppressor miRNA in the pathogenesis of thyroid cancer might vary in different populations based on the frequencies of these variants in each population.

Taken together, miRNAs have critical roles in regulation of thyroid cancer-related signaling pathways. Their availability in body fluids provides the possibility of application of non-invasive sampling in diagnosis of thyroid cancer. A number of miRNAs panels have been shown to be applicable in determination of cancer course and patients prognosis in thyroid cancer. Verification of these results in larger samples sizes of patients from various ethnicities would pave the way for their applications in clinical settings.

## Declaration of competing interest

The authors declare they have no conflict of interest.
